# Impact of CRISPRi-Mediated Titration of *GPD* Genes on the Fermentative Performance of *S. cerevisiae*

**DOI:** 10.1021/acssynbio.5c00316

**Published:** 2025-10-16

**Authors:** João Miguel Spavieri, Thiago Gaspar Inacio, Gustavo Seguchi, Brenda Cristina de Souza, Gonçalo A. G. Pereira, Fellipe de Mello

**Affiliations:** Departamento de Genética, Evolução e Bioagentes, 28132 UNICAMP, Campinas, São Paulo 13086-002, Brazil

**Keywords:** CRISPRi, GPD, glycerol, ethanol, biofuels

## Abstract

Glycerol is one of the main byproducts in ethanol fermentation due to its importance in redox balance and response to osmotic stress in *Saccharomyces cerevisiae*. Since its production diverts carbon from alcohol production, traditional gene-editing methods have been applied to the glycerol synthesis pathway. However, such approaches generate undesirable phenotypes for industrial applications. In the present study, we employed the CRISPR-dCas9 system to moderately downregulate the expression of *GPD1* and *GPD2*, the two main genes involved in this metabolism. *GPD2* gene expression downregulation and a graded reduction in glycerol production after repression of four different target sites in each paralogue were achieved. Employment of the CRISPRi approach for *GPD* gene modulation resulted in higher specific ethanol productivity (SEP) than that of single knockout cells. Targeted modulation in a region −140 basepairs upstream of the transcription start site (TSS) of *GPD1* resulted in a 3% increase in ethanol production compared to the wild type and gpd Δ strains. Such regulation, combined with *GPD2* deletion, revealed the higher SEP among all tested strains. Furthermore, a *GPD1*-modulated strain maintained tolerance to high osmolarity in very high-gravity (VHG) fermentation while maintaining its ethanol production levels above those observed in the control strain.

## Graphical abstract

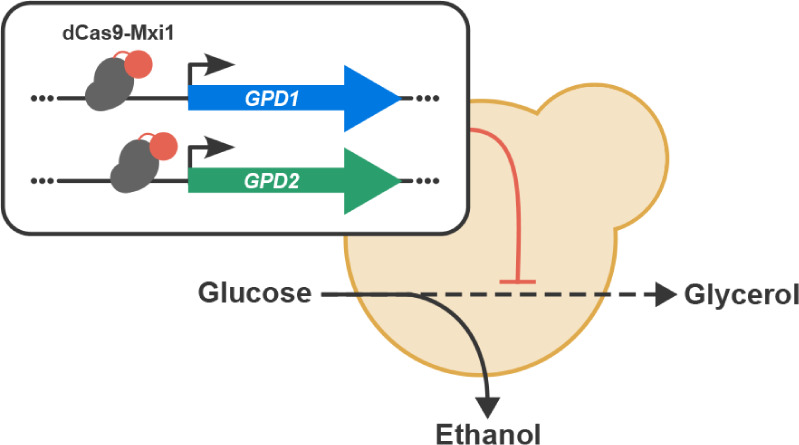

## Introduction

The industrial process for fuel ethanol production is a well-established renewable energy-acquiring bioprocess, producing over 100 billion liters annually since 2017.^1^ Despite the high volumes of the final product, this process still has small profit margins and is greatly affected by feedstock prices, since its costs account for up to 70% of the final production value.^2^ Therefore, small increases in the conversion efficiency of sugar to ethanol greatly impact the economic viability. Currently, the main biofactory for converting sugar into ethanol is the baker’s yeast *Saccharomyces cerevisiae*, which reaches 90%–94% of the theoretical maximum yield for ethanol fermentation in the industrial context.^3^ However, ethanol productivity is directly affected by the yeast’s metabolism. The carbon acquired from sugar can divert from alcohol production mainly to two different and interconnected pathways: biomass production for growth and glycerol production for redox balancing.^4^

The positive net-ATP production established by alcoholic fermentation enables not only yeast cell maintenance but also further growth of the culture. Yet, the cells need to produce other biomolecules to grow. This process reduces the ethanol yield by reducing the total amount of carbon directed to ethanol formation and also generates a surplus of NADH caused by anabolic reactions.^5^ In this context, the glycerol NADH-dependent anabolic pathway is required to balance the concentrations of NADH/NAD^+^, especially in anaerobic conditions, when the electrons of the excess NADH cannot be used in the respiratory chain.^6^ Moreover, glycerol also has an important role in osmotic stress response as a compatible solute, and its production is regulated by the well-known high-osmolarity glycerol (HOG) signaling pathway.^7,8^

There is only one glycerol anabolic pathway known in *S. cerevisiae*, which starts with the conversion of dihydroxyacetone-phosphate (DHAP) to glycerol 3-phosphate (G3P) by NADH-dependent glycerol 3-phosphate dehydrogenase (GPDH) (Figure 1A).^9^ The produced G3P is then dephosphorylated by ATP-dependent glycerol 3-phosphate phosphatase, producing one molecule of glycerol at the expense of ATP and the conversion of NADH to NAD^+^.^10^ The rate-limiting process of this pathway is the conversion of DHAP to G3P by the GPDH enzyme, which is encoded by two paralog genes, *GPD1* and *GPD2*.^11–14^ However, each gene has a particular role in yeast physiology and, hence, a particular regulation of its expression. Particularly, *GPD1* expression is upregulated in the face of osmotic stress, while *GPD2* expression increases during anaerobic growth.^15^ Furthermore, the products of each paralog are differentially involved in other metabolic processes and are present in distinct cellular localizations, besides their main occurrence in the cytoplasm (e.g., Gpd1p in the G3P shuttle).^16–18^ Moreover, the activity of the enzyme encoded by each paralog is also regulated by different post-translational modifications.^19,20^

**Figure 1:**
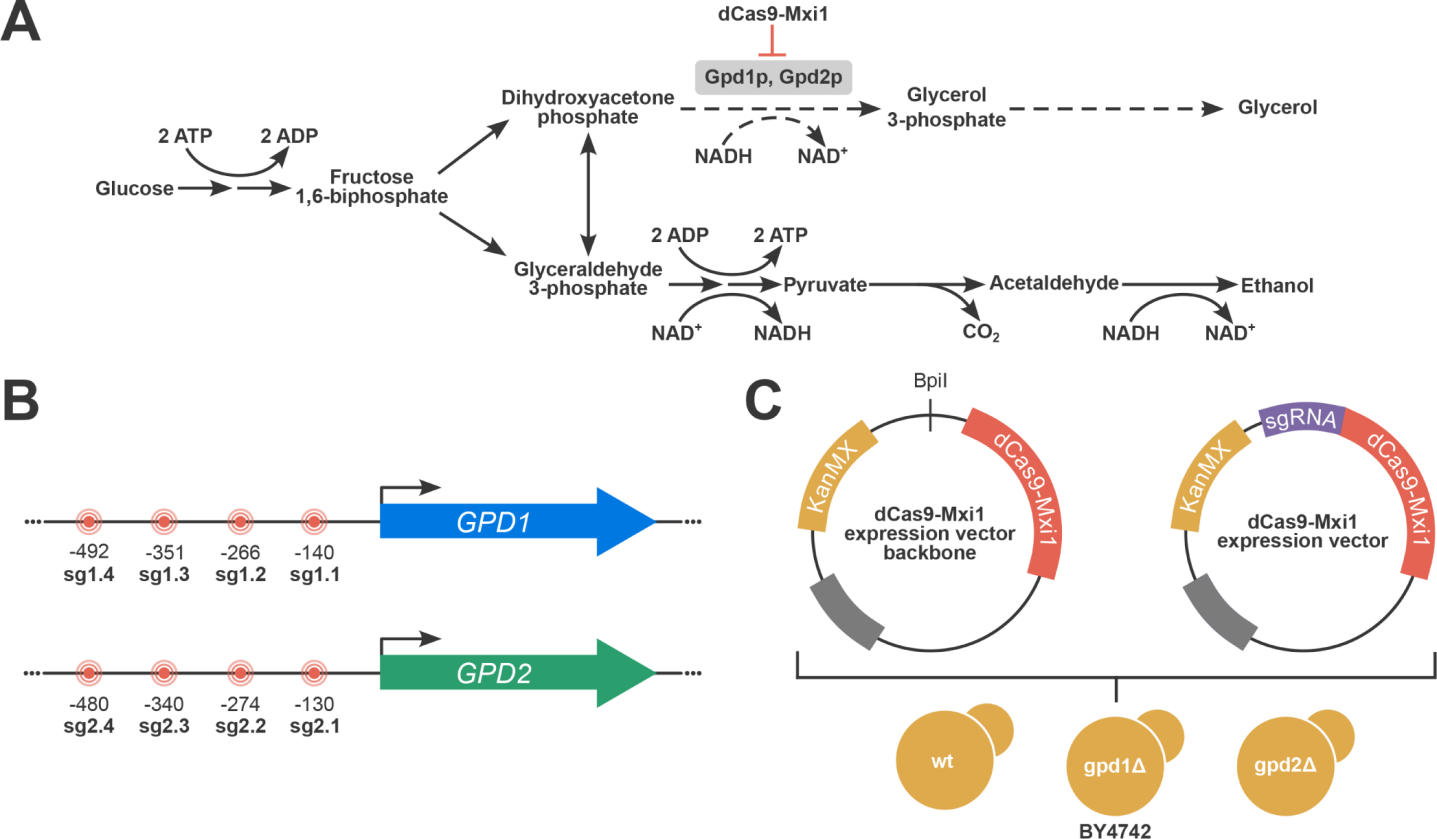
Glycerol pathway regulation by CRISPRi-mediated downregulation of *GPD* genes’ expression levels in *S. cerevisiae* for fermentative traits investigation. A. Glycerol and ethanol anabolic pathways deriving from glucose assimilation indicating the *GPD1* and *GPD2* genes repression by dCas9-Mxi1. B. Schematic representation of the selected target sites’ position in the *GPD* genes promoters with the nomenclature used for the respective sgRNA and their relative distances to the gene TSS. C. The backbone dCas9-Mxi1 expression vector and the dCas9-Mxi1 expression vectors containing the nine different sgRNA coding sequences were transformed into three BY4742 background strains.

Deletion of both *GPD1* and *GPD2* was already applied to enhance the carbon flux in ethanol production. The deletion of each *GPD* paralog alone resulted in an overall decrease in glycerol yield with increased ethanol production in anaerobic and quasi-anaerobic conditions.^21–25^ However, because of the physiological importance of each paralog, the engineered strains showed undesirable growth phenotypes: *GPD1* deletion resulted in an extremely osmosensitive strain^11,12,26^ while gpd2Δ cells had their growth affected in anaerobic cultures.^21–23^ Double knockout strains showed no production of glycerol and increased ethanol yield but slower growth, along with all the undesirable phenotypes observed after *GPD1* and *GPD2* individual knockouts.^15,22,23,26^ Seeking a balance between the unattractive traits obtained with the binary gene editing methods and an increase in ethanol yield, the reduction of glycerol production without abolishing *GPD* gene expression appears as an interesting approach for metabolism rewiring. Until now, such attempts have been described only through promoter engineering of *GPD1* and *GPD2*.^26–28^

While promoter selection is a well-established tool for regulating gene expression in genetic engineering, its flexibility is limited and largely confined to a small set of well-characterized sequences. Meanwhile, the CRISPR-dCas9 system has been successfully applied for gene expression modulation in *S. cerevisiae*.^29–31^ This approach consists of targeting the mutated and noncatalytic endonuclease dCas9, alone or fused with activators or repressors, to a gene of interest using a guide RNA. As an outcome, dCas9 regulates the targeted gene expression without cleavage of the DNA molecule. This regulation can result in either activation (CRISPRa) or inactivation (CRISPRi), according to the target site and the regulatory complex fused with dCas9.^29^ In *S. cerevisiae*, dCas9 fused with the regulatory complex Mxi1 is mostly used for CRISPRi, with up to a 53-fold reporter gene activity repression, compared to 18-fold with solely dCas9.^29^ However, the regulation strength is very location-dependent, with maximal repression when sites in the −200 bp to the TSS window are targeted.^30^

Thus, CRISPRi appears as an attractive strategy to fine-tune glycerol production without greatly affecting the physiology of yeast growth in the alcoholic fermentation process. With this purpose, we constructed laboratory *S. cerevisiae* strains with distinct levels of glycerol production using a dCas9-Mxi1 expression vector targeting the *GPD* genes in four different target sites. Besides, we also combined the deletion of the nonregulated paralog with CRISPRi differential repression. Moreover, we tested concurrent *GPD1* and *GPD2* repression by dCas9-Mxi1 in a single strain. Finally, we analyzed the *GPD* genes’ expression, growth, glycerol production, ethanol production, and osmotolerance by the constructed strains.

## Materials and Methods

### Strains, Plasmid, and Media

All the engineered *S. cerevisiae* strains constructed in the present study were derived from the laboratory strain BY4742 (*MATα his3Δ1 leu2Δ0 lys2Δ0 ura3Δ0*).^32^ The yeast strains were grown in YPD (1% yeast extract, 2% peptone, and 2% glucose) at 30 °C, with the addition of Geneticin (G418, 200 μg/mL) or hygromycin (300 μg/mL) when required for selection or plasmid maintenance. Stock cultures were maintained at −80 °C in YPD with 25% glycerol. Plasmid amplification was performed in the DH5α *Escherichia coli* strains (Thermo Fisher Scientific). All the *E. coli* strains transformed with plasmids were grown in Luria–Bertani (LB) medium (0.5% yeast extract, 1% peptone, 1% NaCl) supplemented with kanamycin (50 μg/mL) at 37 °C. The EC2_3_dCas9_Mxi1_sgRNA dCas9-Mxi1 expression vector (Addgene plasmid #163708) created by Cámara et al.^33^ was used to construct all the plasmids used in this study.

### Selection of Target Sites

The target site selection was carried out using the Yeast CRISPRi webtool^30^ to avoid sites with high nucleosome occupancy and low chromatin accessibility. Sequentially, the single-guide RNAs (sgRNAs) for the regulation of the chosen sites were also analyzed through the CRISPR-ERA tool^34^ for specificity evaluation. All of the target sites were set upstream of the TSS with an approximate distance of 100 base pairs from each other when targeting the same promoter. The eight sgRNAs targeting the selected sites are herein referred to according to the targeted paralog and the proximity to the gene TSS. For *GPD1*, sgRNAs are described as sg1.1 to sg1.4, and for *GPD2*, as sg2.1 to sg2.4. The first number, before the dot, indicates which *GPD* gene the sgRNA is relative to, and the second is related to the target site location, with 1 representing the closest TSS and 4 representing the most distant. All sgRNAs selected for this study are available in Table S1.

### CRISPRi Vector Assembly

The construction of the vectors containing the sgRNAs for each CRISPRi target site was carried out by homologous recombination directly in *S. cerevisiae*, as previously described by Cámara et al.^33^ In short, the dCas9-Mxi1 expression vector was linearized using the BpiI (Thermo Fisher Scientific) restriction enzyme, following the manufacturer’s instructions. Complementary single-stranded oligonucleotides (ssOligos) containing the 20-base pair (bp) sgRNA sequence flanked by a 40 bp sequence with homology to the recombination site in the plasmid at both ends were synthesized for each sgRNA sequence. The two ssOligos regarding the same sgRNA sequence were then hybridized by heating to 95 °C for 6 min and subsequent cooling of −1 °C/min until 25 °C was reached and maintained for 5 min. Finally, 100 ng of the linearized plasmid and 5 μL of the hybridization product containing the double-stranded oligonucleotide (dsOligos) with the sgRNA sequence were transformed into yeast according to Gietz and Schiestl.^35^

The multiplex plasmid containing one sgRNA for each *GPD* paralog was constructed by *in vivo* homologous recombination of fragments from two dCas9-Mxi1 expression vectors. The dCas9-Mxi1 and sgRNA 2.1 coding sequences digested with XbaI (Thermo Fisher Scientific) and *Pst*I (New England Biolabs) restriction enzymes were transformed into BY4742 together with the sgRNA 1.1 coding sequence and all of the other components of the backbone plasmid. Both the sgRNA 1.1 coding sequence and the backbone plasmid components were amplified by polymerase chain reaction (PCR) from the previously cloned dCas9-Mxi1 sg1.1 expression vector, with primers containing homologous ends. The transformation was performed using van Leeuwen et al.’s^36^ method. The transformed strains were incubated in solid YPD supplemented with Geneticin at 30 °C for 72 h for selection. The correct recombination of the multiplex plasmid was verified by Sanger sequencing, while the other plasmids were verified via colony PCR. The correctly cloned plasmids were all extracted from yeast according to the Sobanski and Dickinson^37^ method for further transformation in *E. coli* by electroporation.^38^ All primers and plasmids used and cloned in the study are described in Tables S2 and S3.

### Strains Construction

The gpd1Δ and gpd2Δ strains were constructed by knock-in of a hygromycin resistance expression cassette (hphMX6) PCR-amplified from pAG32 ^39^ with primers containing ends with homology outside the *GPD* genes open reading frames (ORF). All primers used are described in Table S2. The transformed strains were incubated in solid YPD supplemented with hygromycin at 30 °C for 72 h for selection. The correct recombination was verified by phenotype and colony PCR. For gene modulation, all strains (BY4742, BY4742 gpd1Δ, and BY4742 gpd2Δ) were transformed with the dCas9-Mxi1 expression vectors according to Gietz and Schiestl.^35^ The BY4742 strain was transformed with the expression vectors containing sgRNA targeting 4 different sites in *GPD1* and 4 in *GPD2*, and the multiplex plasmid. In turn, BY4742 gpd1Δ was transformed only with vectors containing the 4 sgRNAs for *GPD2* modulation, while BY4742 gpd2Δ was transformed with the plasmids carrying the 4 sgRNAs targeting *GPD1*. Strains were also transformed with the dCas9-Mxi1 expression vector backbone, i.e., without any sgRNA coding sequence, as a control. All dCas9-Mxi1 transformed strains were collected in YPD supplemented with Geneticin. A list of all strains used in this study is available in Table S4.

### Fermentation Assays

All of the engineered strains were cultivated in triplicate during the fermentations for growth, glycerol production, and ethanol production evaluations. The standard fermentation assays were carried out in 25 mL Schott flasks containing 20 mL of YPD 5% glucose supplemented with Geneticin at 30 °C and 200 rpm in a shaking incubator. The cultures were inoculated at an optical density (OD_600_) of 1 from inocula cultivated in YPR (1% yeast extract, 2% peptone, 2% raffinose) supplemented with Geneticin at 30 °C and 250 rpm. Since raffinose is a nonfermentable carbon source, YPR was used for yeast propagation prior to the fermentation to ensure fermentation started only when the cells were transferred to YPD. With this approach, we could minimize possible effects of *GPD* genes modulation in the inocula’s cells physiology. The fermentation was carried out for 36 h, with sample collection right after the inoculation (0 h), and after 12, 18, 24, and 36 h. The very high-gravity (VHG) fermentation assay was carried out using the same inoculum conditions as those used for the standard fermentation. The inocula were used at an OD_600_ of 1 in 25 mL Schott flasks containing 20 mL of YPD 25% glucose supplemented with Geneticin at 30 °C and 200 rpm in a shaking incubator. The fermentation was carried out for 72 h, with sample collection after 24, 48, and 72 h. OD_600_ of the samples collected from both standard and VHG fermentation was measured using an Ultrospec 2000 (Pharmacia Biotech) spectrophotometer after proper dilution.

### RT-qPCR Assays

For RT-qPCR assays, strains were propagated in YPR supplemented with Geneticin at 30 °C and 250 rpm. The cultures for sampling were inoculated in 20 mL of YPD 5% glucose supplemented with Geneticin in 25 mL Schott flasks at 30 °C and 200 rpm with an initial OD_600_ of 1. Samples were collected after 24 h, centrifuged, separated from the medium, and snap-frozen in liquid nitrogen. RNA was extracted using a phenol–chloroform method,^40^ while cDNA synthesis was carried out with PrimeScript RT Reagent Kit (Takara). For all of the samples, 10 ng of the generated cDNA was used for qPCR using PowerTrack SYBR Green Master Mix (Thermo Fisher Scientific) in the StepOnePlus Real-Time PCR System (Applied Biosystems). The primers used for qPCR assays are listed in Table S2. All the reactions were set in quadruplicates, and a melting curve analysis was carried out to verify nonspecific amplification and primer-dimer formation. The ΔΔCt method was used to quantify *GPD* expression in CRISPRi-modulated and single-knockout strains in relation to the control strain, with *ACT1* as a reference gene.

### Analytical Methods

The collected samples from fermentation were used for glucose, glycerol, and ethanol concentration evaluation by high-performance liquid chromatography (HPLC) after dilution (1:15) in ultrapure water (Milli-Q) and filtration. The procedure was performed in Alliance HPLC System (Waters) using Aminex HPX-87H (300 mm × 7.8 mm, Bio-Rad) ion-exchange column with a refractive index detector (Waters 2414 RI detector) at 410 nm. The column was maintained under 50 °C with a 0.6 mL/min flow rate for 30 min.

### Trait Evaluation in Solid Medium

For the growth assay in osmotic stress, all of the strains were grown overnight in conventional YPD medium with Geneticin at 30 °C and 250 rpm. Then, OD_600_ was adjusted to 1 and a 1:10 serial dilution was prepared for each strain. 5 μL of each dilution was spotted onto a solid medium for all the strains. The medium used for osmosensitivity trait evaluation was a glucose-rich YPD (25% glucose) supplemented with Geneticin. The plated cells were then incubated at 30 °C for 72 h, and imaging was carried out in Gel Doc XR+ (Bio-Rad).

### Statistical Analysis

The statistical analyses were carried out using one-way analysis of variance (ANOVA) and Tukey’s test when data residuals were normally distributed, while non-normally distributed data was analyzed by Kruskal–Wallis and Dunn tests. Pearson correlation was applied to investigate correlations between the two variables. Normality and homoscedasticity of the data were analyzed using the Shapiro–Wilk test for the data residuals and Levene’s test, respectively. All of the statistical analyses were performed in R, considering a level of significance of 0.05.

## Results and Discussion

### CRISPRi sgRNA Design

Aiming to better understand the glycerol metabolism for further improvement of fermentative traits in yeast, the impact of different levels of *GPD* expression repression was investigated. For such, dCas9-Mxi1 expression vectors were cloned into strain BY4742: four containing sgRNAs coding sequences targeting distinct sites in the *GPD1* promoter, four for the *GPD2* promoter, and one carrying sgRNAs targeting both *GPD1* and *GPD2* (Figure 1B). The multiplex plasmid included sgRNAs targeting the sites closest to the TSS of both genes.

Chromatin accessibility and nucleosome occupancy are the main drivers of CRISPRi effectiveness, therefore, the targeting site selection was based on these two factors, together with sgRNA specificity.^30^ On *S. cerevisiae*’s gene promoters, the chromatin accessibility is usually higher inside the ORFs, while the nucleosome occupancy is lower, especially between the TSS and −200 bp.^41^ For this reason, all of the selected targeting sites are located upstream of the TSS. Furthermore, the repression strength of dCas9-Mxi1 regulation varies according to the targeted site. Aiming to achieve a gradient of repression strength, the target sites were chosen to be approximately 100 bp distant from each other.

All of the sgRNA sequences were cloned into the dCas9-Mxi1 expression backbone vector, and the plasmids were then transformed into three different strains: BY4742, BY4742 gpd1Δ, and BY4742 gpd2Δ (Figure 1C). The multiplex plasmid, carrying both sg1.1 and sg2.1 sgRNA sequences, was transformed only into BY4742. Also, to reduce the chance of plasmid presence bias, the BY4742, BY4742 gpd1Δ, and BY4742 gpd2Δ strains used in the experiments were transformed with the vector backbone.

### Impact of CRISPRi on *GPD* Expression Levels

Interested in better understanding the differential profiles of each strain after CRISPRi modulation, we analyzed the expression levels of *GPD1* and *GPD2* in all eight strains. We also analyzed the single-knockout strains’ expression to validate the deletions and our qPCR method (Figure 2). The control strain was used to perform a relative analysis of gene expression, and the *ACT1* gene was selected as a reference gene.

**Figure 2:**
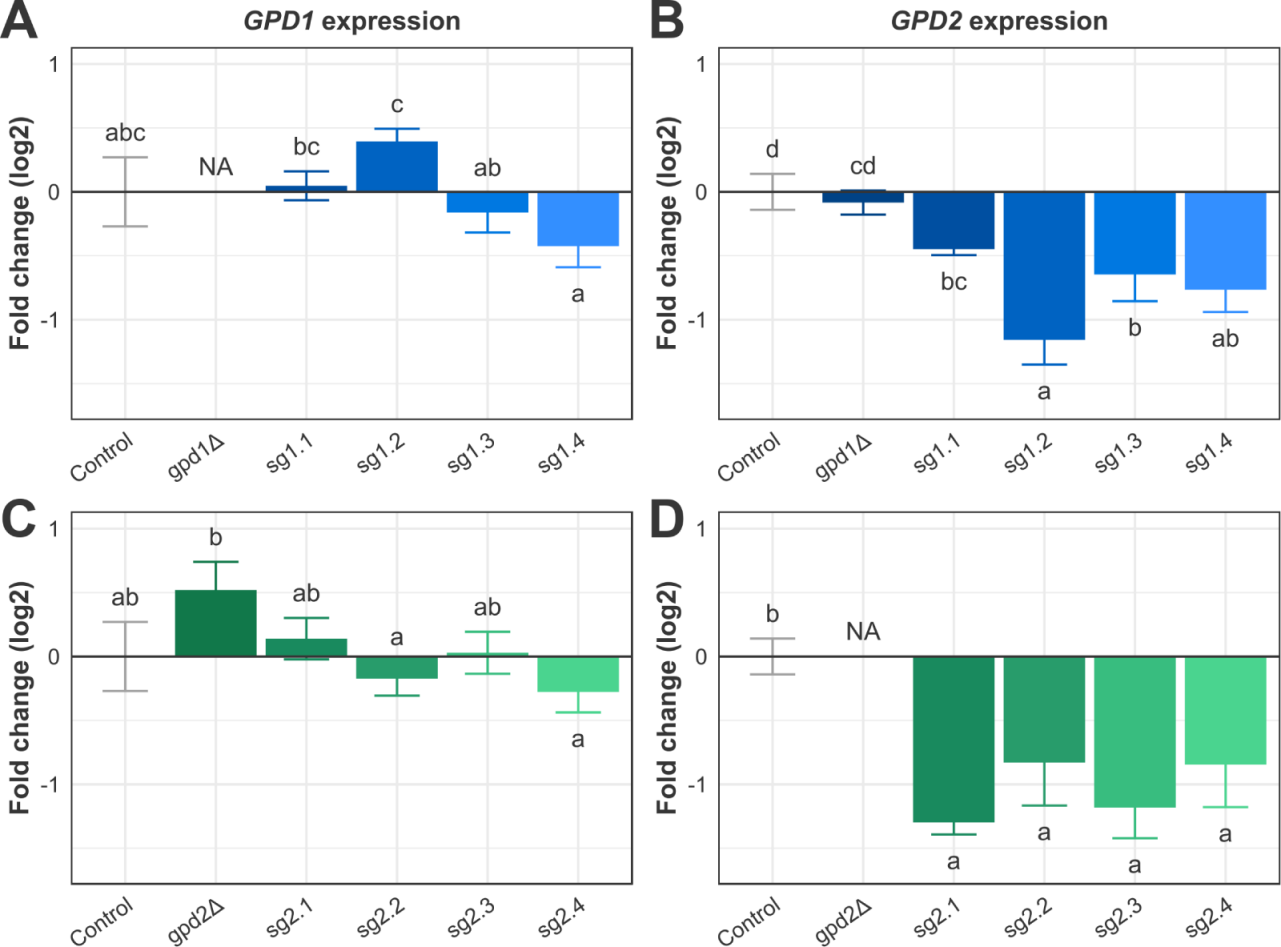
Relative expression levels of *GPD1* and *GPD2* after knockout or CRISPRi modulation. A. *GPD1* expression levels in the *GPD1*-modulated strain set and the gpd1Δ strain. B. *GPD2* expression levels in the *GPD1*-modulated strain set and the gpd1Δ strain. C. *GPD1* expression levels in the *GPD2*-modulated strain set and the gpd2Δ strain. D. *GPD2* expression levels in the *GPD2*-modulated strain set and the gpd2Δ strain. NA: lack of amplification in qPCR assays.

The single-knockout strains did not express their respective deleted genes, validating their phenotypes and the specificity of the used primers. Moreover, they presented distinct profiles of gene expression compensation (Figure 2B,C). The gpd1Δ strain did not significantly alter *GPD2* expression to compensate for the lack of *GPD1* transcripts. In turn, *GPD1* expression mildly increases in gpd2Δ cells. The *GPD1* differential compensation behavior indicates that its expression might be responsive to glycerol production decreases and is consistent with literature results.^15,21,23^ In turn, *GPD2* is known to respond only to the intracellular NAD^+^/NADH ratio, which confers its role in anaerobic growth.^15^

Surprisingly, the *GPD1*-modulated strains showed minor alterations in *GPD1* expression levels and significant repression of *GPD2*, especially in the sg1.2 strain (Figure 2A and 2B). This result may indicate the intricate nature of *GPD1* regulation in *S. cerevisiae* or an off-target activity, even with a low similarity between the *GPD1*-directed sgRNA sequences and the *GPD2* promoter. However, the *GPD1*-modulated strains showed distinct *GPD* expression profiles among them. Aiming to investigate the impact of such alterations in gene expression on fermentative performance, we decided to continue analyzing these strains.

Regarding the *GPD2*-modulated strains, *GPD2* mRNA levels decreased to approximately half of the control strain level, without large differences in repression strength among the strains (Figure 2D). Interestingly, the *GPD1* expression was not altered by CRISPRi in *GPD2*, which highlights a distinct pattern of gene expression compensation after controlled repression and complete knockout (Figure 2C).

The analysis of each *GPD* gene expression profile after CRISPRi underscores that dCas9-based fine-tuning approaches are highly dependent on the targeted genes, and its modulation should be evaluated on a case-by-case basis.^43^ To obtain further information about the consequences of the modulated *GPD* expression patterns, we assessed the phenotypes of each strain. Therefore, we performed a small-scale batch fermentation to analyze glycerol, ethanol, and biomass production.

### *GPD* CRISPRi-Modulated Strains’ Fermentative Profiles

The *GPD* genes’ CRISPRi-directed regulation effects were investigated in a small-scale batch fermentation under semiaerobic conditions. The YPD medium was chosen for growth with a glucose concentration higher than usual (50 g/L), although not capable of inducing osmotic stress.^26^ These conditions were selected to avoid the induction of stress responses that could alter the yeast metabolism and ensure that the results are due to the modulation. The time course graphs of glucose consumption, biomass formation, glycerol production, and ethanol production during the fermentation are shown in Figure S1. The glycerol and ethanol yield values are also displayed in Table S5. Because all the tested strains could consume all the glucose after 24 h of fermentation without ethanol and glycerol consumption, this time point was selected for further analysis in the study (Figure 3). Biomass values, final glycerol and ethanol concentrations, and specific ethanol productivity (SEP) are also listed in Table S6. A complete carbon recovery measurement was not performed due to the use of a rich medium.

**Figure 3:**
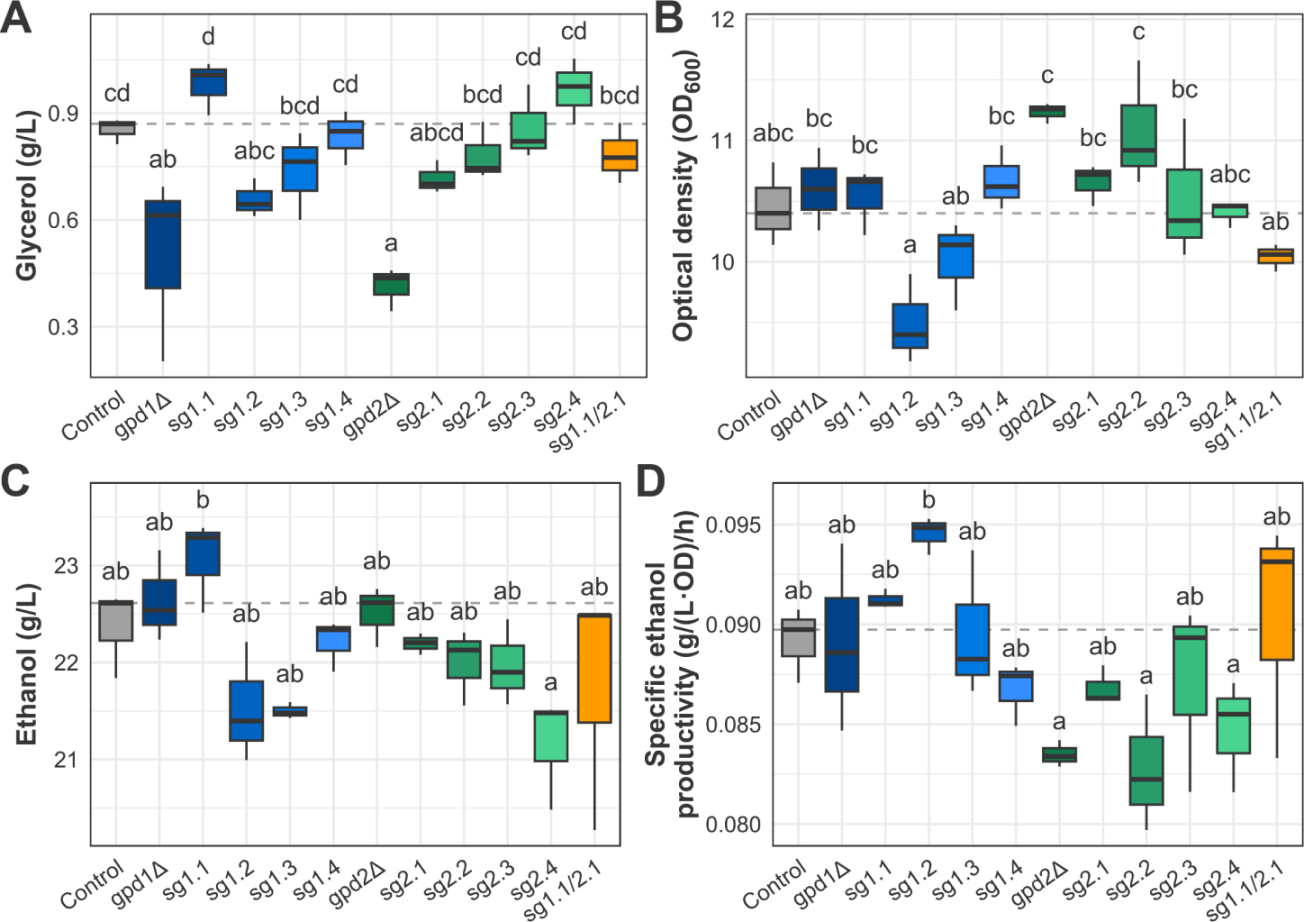
Fermentative profile of the CRISPRi-modulated, the *GPD* genes knockout, and the control strains after 24 h of semianaerobic fermentation in YPD 5% glucose indicates target-specific effects of CRISPRi modulation on metabolites and biomass production. A. Glycerol final concentration for each of the tested strains. B. Biomass formation of the tested strains according to the optical density at 600 nm of the culture. C. Ethanol final concentration of the tested strains. D. Specific ethanol productivity of each strain. Boxplots represent minimum, maximum, quartile, and median values, and the letters indicate statistically significant differences between strains.

The *GPD* single-deletion strains showed significantly lower production of glycerol when compared to the control, with 41.1% and 51.6% less glycerol produced by gpd1Δ and gpd2Δ, respectively (Figure 3A). This reduction was accompanied by a carbon flux drift to biomass production in the gpd2Δ strain, which grew 7.5% more than the control (Figure 3B). In turn, the *GPD1* deletion caused a minor increase in biomass formation (Figure 3B). Moreover, the single-deletion strains did not show significantly higher ethanol final concentrations when compared to the control strain (Figure 3C), as similarly reported by Björkqvist et al.^22^ Regarding the SEP, both single-deletion strains presented values below the control strain.

In the CRISPRi-modulated strains, we observed target-site-dependent effects in the fermentative profile of each strain. Except for the sg1.1 strain, the strains produced the same amount of glycerol or less than the control. Among the eight constructed strains for a single gene target, sg1.4, sg2.3, and sg2.4 showed a final glycerol concentration similar to that of the control strain. In turn, strains sg1.2, sg1.3, sg2.1, and sg2.2 produced significantly less glycerol. The biggest decrease in glycerol production was observed for the sg1.2 strain, which produced 26% less than the control (Figure 3A). The sg2.1, sg1.3, and sg2.2 showed 16.2%, 13.8%, and 8.4% reductions in final glycerol concentrations, respectively. Contrary to the expected outcome, *GPD1* repression closest to its TSS resulted in higher glycerol production levels.

The regulation of *GPD* genes expression by dCas9-Mxi1 also altered biomass formation for most of the tested strains (Figure 3B). Except for sg2.4, all of the strains showed variation in growth compared to the control. In general, the *GPD2*-modulated strains grew more, while the *GPD1*-modulated strains presented more varied phenotypes. The sg1.2 and sg1.3 strains grew 9.2% and 4.2% less than the control, respectively. In turn, sg1.1 and sg1.4 showed minor increases in biomass formation.

When analyzing ethanol final concentration levels, only two strains presented statistically significant differences compared to the control (Figure 3C). Namely, sg1.1 produced roughly 3% more ethanol, while sg2.4 produced 5.4% less ethanol than the control. The increased ethanol concentration by the sg1.1 strain was interestingly obtained along with a glycerol concentration increase. Although unexpected, this promising result indicates more complex dynamics in central carbon metabolism during semianaerobic growth. However, we notice a general trend of a lower final ethanol concentration after CRISPRi. Even though decreased glycerol production was observed, such reduction did not necessarily lead to higher ethanol final concentration levels due to carbon flux diversion to biomass formation or growth limitation.

Considering *GPD* modulation-directed changes in biomass formation, we calculated the SEP to shed light on ethanol production accounting for the differential growth of each strain (Figure 3D). The *GPD1*-modulated strains presented distinct SEP values, with the sg1.1, sg1.2, and sg1.3 strains above the control strain level and the gpd1Δ strain. The sg1.2 strain, which had the most hindered growth among all of the strains, showed a 6.7% increase in relation to the control, presenting the highest SEP overall. In turn, all the *GPD2*-modulated strains remained below the control level, corroborating the observed higher growth throughout this set of yeast. Notably, the sg2.1 strain presented SEP above that obtained by the null *GPD2* cells. Overall, CRISPRi regulation of both GPD genes resulted in higher SEP relative to knockout strains, suggesting that such an approach induces more efficient ethanol production relative to biomass.

Aiming to obtain further levels of repression, we also analyzed the fermentation performance of a strain carrying one plasmid with two distinct sgRNA coding sequences, one targeting *GPD1* and one targeting *GPD2*. The chosen sgRNAs were sg1.1 and sg2.1, which target the sites closest to each gene ORF. However, the sg1.1/2.1 strain produced only 8.2% less glycerol than the control, similar to the values observed for the strains with just one targeted site (Figure 3A). The strain also exhibited a minor decrease in growth compared to that of the control (Figure 3B). Regarding ethanol production, the final ethanol concentration and the SEP showed no statistically significant alteration (Figure 3C and D). The combined *GPD* gene repression did not result in an amplified reduction of glycerol production, probably due to the selected target sites. As discussed, the sg1.1 strain showed more glycerol production than the control strain. Therefore, the sg1.1/2.1 glycerol production can be interpreted as a balance between a glycerol production increase caused by repression in the sg1.1 target site and a decrease derived from repression in the sg2.1 site.

Interestingly, the glycerol production values obtained from the small-scale batch fermentation do not significantly correlate with the gene expression levels, nor for *GPD1* nor *GPD2*. For instance, even though the *GPD1*- and *GPD2*-modulated strains showed lower glycerol concentrations produced by strains carrying target sites closer to the *GPD* TSSs, except for the sg1.1 strain, we could not observe a significant correlation between glycerol concentration and the modulated *GPD* expression levels (Figure 4A,D). Moreover, it is possible to observe a trend of negative correlation between *GPD1* expression in the *GPD2*-modulated strains (Figure 4B) and positive correlation between *GPD2* expression in the *GPD1*-modulated strains (Figure 4C). The discrepancy between phenotypic attributes and expression levels may be caused by the GPDH activity complex regulatory network, which is strongly affected by translational and post-translational mechanisms, e.g., differential phosphorylation and translational regulation mechanisms.^19,20,43^

**Figure 4:**
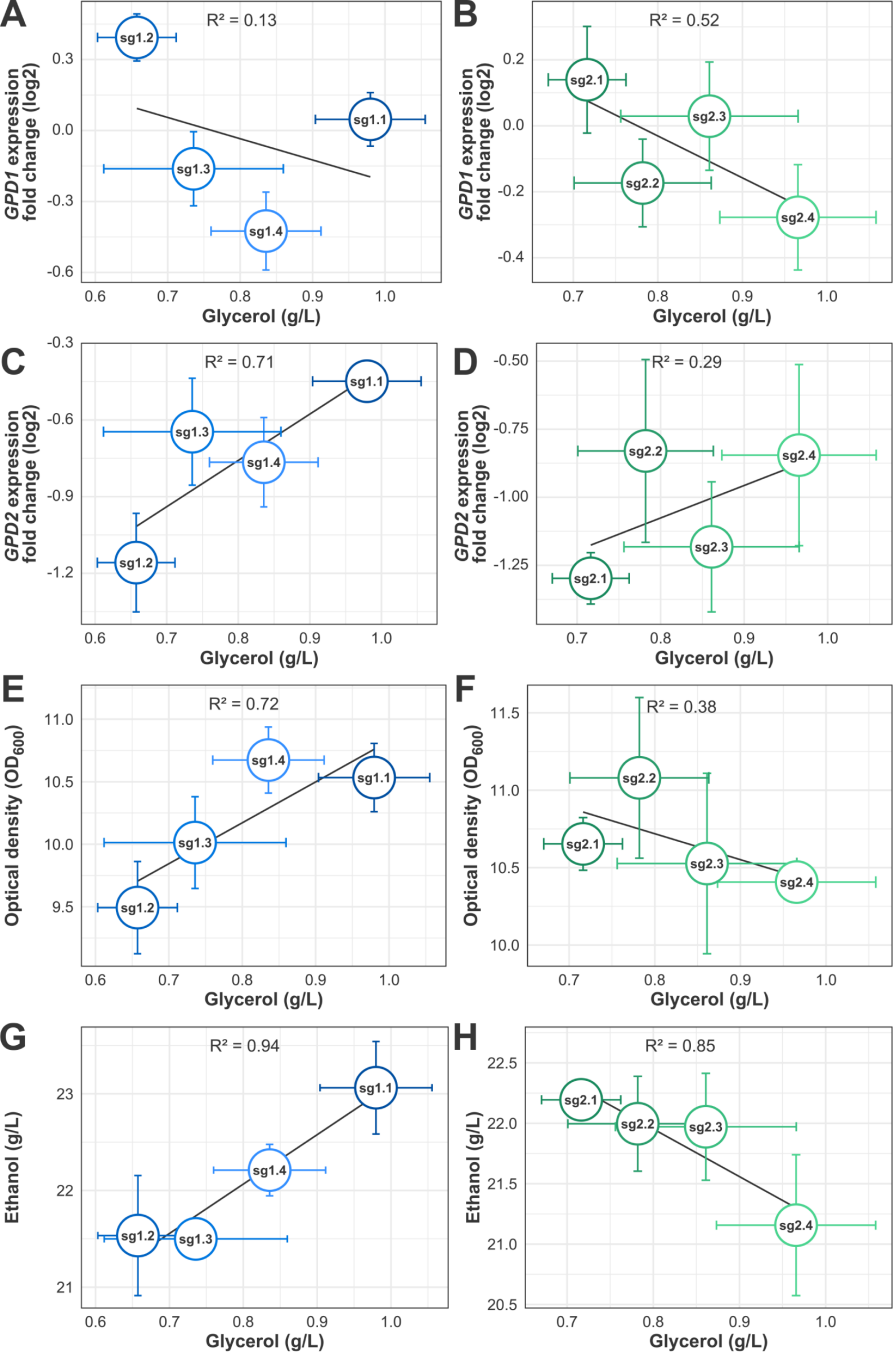
Correlations between the gene expression data and fermentation variables after 24 h of fermentation indicate paralog-specific effects for the *GPD* genes undergoing CRISPRi-mediated glycerol production decrease. A. Correlation between *GPD1* expression fold-change and glycerol production in *GPD1*-modulated strains. B. Correlation between *GPD1* expression fold-change and glycerol production in *GPD2*-modulated strains. C. Correlation between *GPD2* expression fold-change and glycerol production in *GPD1*-modulated strains. D. Correlation between *GPD2* expression fold-change and glycerol production in *GPD2*-modulated strains E. Correlation between culture optical density mean values (at 600 nm) and mean glycerol production for each *GPD1*-modulated strain. F. Correlation between culture optical density mean values (at 600 nm) and mean glycerol production for each *GPD2*-modulated strain. G. Correlation between mean ethanol production and mean glycerol production for each *GPD1*-modulated strain. H. Correlation between mean ethanol production and mean glycerol production for each *GPD2*-modulated strain.

CRISPRi-modulated strains presented paralog-specific correlations between the glycerol final concentration and ethanol and biomass formation. The *GPD1* modulation led to a positive correlation between glycerol production and biomass formation (*R* ^2^ = 0.72), as well as between glycerol production and ethanol production (*R* ^2^ = 0.94) (Figure 4E and G). Therefore, biomass formation and ethanol production follow glycerol production levels when *GPD1* is modulated. Indeed, the sg1.1 and sg1.2 strains, respectively, the greatest and the least *GPD1*-modulated glycerol producers, are also the greatest and the least ethanol and biomass producers among the *GPD1*-modulated strains.

Although unexpected, this result might be linked to *GPD1*’s role in redox balancing during respiration. The Gpd1p integrates the G3P shuttle, a metabolic pathway important for mitochondrial NAD^+^/NADH balance during respiratory growth of *S. cerevisiae*.^11,17^ Since all glucose was depleted after 18 h of fermentation, the only carbon sources available for growth at 24 h were nonfermentable, such as acetate. Therefore, we hypothesize that the *GPD1*-modulated strains had their growth coupled to glycerol production in the tested conditions, because of glycerol synthesis’s role in the mitochondrial matrix’s redox balance. Then, in the context of reduced production of glycerol, fewer cells are present in the medium, and the reduced growth is accompanied by a proportional decrease in ethanol production. However, at the cellular level, the ethanol synthesis tended to be prioritized by the *GPD1*-modulated strains, as indicated by the SEP values.

In contrast, since the *GPD2* product is not involved in any known exclusive physiological role under the tested conditions, modulation of *GPD2* pointed the strains’ fermentative profile in the opposite direction. The glycerol production levels in the *GPD2*-modulated strains negatively correlated with biomass formation (*R* ^2^ = 0.38) and ethanol production (*R* ^2^ = 0.85) (Figure 4F and H). Within this set of strains, the decrease in glycerol production led to a carbon drift to the competitive pathways of biomass formation and ethanol production. Since most of the *GPD2*-modulated strains grew more than the control, while none produced more ethanol, yeast metabolism prioritized diverting the carbon to biomass formation. Such prioritization could also be noted by analyzing their overall lower SEP.

### *GPD* Concurrent Deletion and CRISPRi Modulation Drastically Reduce Glycerol Production

Aiming to boost the drift of the carbon flux to the alcoholic fermentation pathway, we also investigated the impact of concurrent deletion and CRISPRi modulation of the *GPD* genes. With this purpose, strains with one *GPD* paralog deleted were transformed with dCas9-Mxi1 expression vectors containing sgRNAs coding sequences targeting the nondeleted paralog in the same selected four target sites for each gene.

Therefore, batch fermentation was performed with these strains under the same conditions previously described. Similarly, all the strains were able to completely consume the glucose after 24 h, although showing distinct growth, glycerol final concentration, ethanol final concentration, and SEP (Figure 5). The time course graphs of growth, glucose consumption, glycerol production, and ethanol production throughout the fermentation, together with the glycerol and ethanol yield values, are available in Figure S2 and Table S5. Biomass values, glycerol final concentrations, ethanol final concentrations, and SEP are also listed in Table S6.

**Figure 5:**
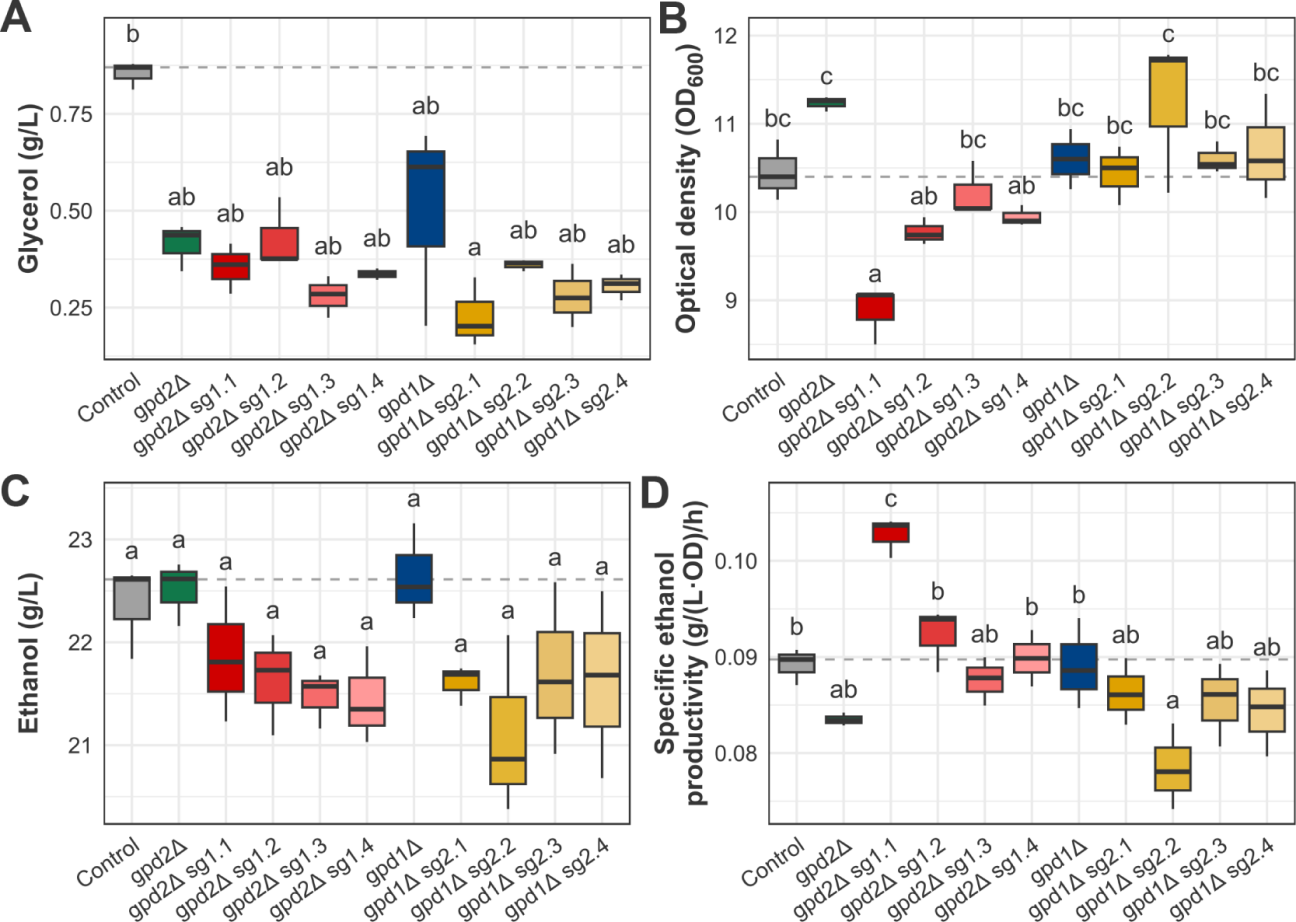
Fermentative profile of the strains carrying both *GPD* single deletion and dCas9-Mxi1 expression vector after 24 h of semianaerobic fermentation in YPD 5% glucose indicates the approach’s significant impacts on glycerol production and SEP. A. Glycerol final concentration for each of the tested strains. B. Biomass formation of the tested strains according to the optical density at 600 nm of the culture. C. Ethanol final concentration of the tested strains. D. Specific ethanol productivity of each strain. Boxplots represent minimum, maximum, quartile, and median values, and the letters indicate statistically significant differences between strains.

The concurrent deletion and CRISPRi modulation of the *GPD* genes resulted in a stronger reduction of glycerol production when compared to sole CRISPRi modulation (Figure 5A). Except for the gpd2Δ sg1.2 strain, all of the tested strains produced less glycerol than the *GPD* single-deletion strains, gpd1Δ, and gpd2Δ. The biggest reduction in glycerol production was observed for the gpd1Δ sg2.1, which produced 73% less glycerol than the control strain and 54.7% less than gpd1Δ. Thus, combining CRISPRi and gene deletion is an alternative to obtain reductions in glycerol yield beyond the single deletion without abolishing glycerol production.

Regarding biomass production, significant differences were observed for only a few strains (Figure 5B). The most hampered growth was observed for the gpd2Δ sg1.1 strain, which grew 15% less than the control. In turn, the strain with the higher growth levels, gpd1Δ sg2.2, grew 7.6% more than the control. Despite the minor overall decrease in the ethanol final concentration, the SEP reflected the biomass production profiles (Figure 5C and D). The gpd2Δ sg1.1 and gpd1Δ sg2.2 strains were the best and worst ethanol producers compared with the other strains carrying concurrent *GPD* deletion and CRISPRi modulation, respectively. Strain gpd2Δ sg1.1 displayed a remarkable 15.7% increase in SEP, representing the best result within all tested strains. Notably, all gpd2Δ cells with *GPD1* modulation presented higher SEP than did the single *GPD2* knockout strain. Once again, this observation reinforces that such a strategy produces strains that effectively produce ethanol over biomass.

The concurrent *GPD* deletion and CRISPRi modulation strategy was not effective in obtaining higher ethanol final concentrations. However, the SEP increase obtained for the gpd2Δ sg1.1 stimulates further investigations, such as the application of a more concentrated inoculum of this strain in future fermentations. Moreover, we succeeded in reducing glycerol production beyond the levels obtained with the gpd1Δ and gpd2Δ strains. Together with the solely CRISPRi-modulated strains’ fermentation, this strategy allowed us to take a step further in the understanding of the intricate glycerol pathway relations with biomass and ethanol production.

### CRISPRi *GPD* Modulation Effects on Osmotolerance Are Strain-Dependent

Glycerol is the main osmolyte produced by *S. cerevisiae* cells in osmotic stress, and its production is especially important for cell viability in these conditions.^44^ The glycerol production in response to high osmolarity is dependent on the induction of *GPD1* expression by the high-osmolarity glycerol (HOG) signaling pathway.^7,8^ Traditional gene-editing approaches for glycerol yield decrease produced strains sensitive to high osmolarity when directed to *GPD1*.^11,12,26^ Since industrial fermentation processes usually utilize substrates with high sugar concentrations, high-osmolarity-sensitive phenotypes must be avoided. Therefore, we decided to investigate whether glycerol production titration by dCas9-Mxi1-mediated repression would lead to an osmo-sensitive phenotype, especially when targeting *GPD1*. For such analysis, a drop assay was carried out with serial dilutions of all the engineered strains spotted in glucose-rich (25% glucose) solid YPD medium for 72 h (Figure 6). The same strains were also spotted in solid YPD with 2% glucose for 48 h as a control (Figure S3), where it can be observed that growth was similar to all yeasts.

**Figure 6:**
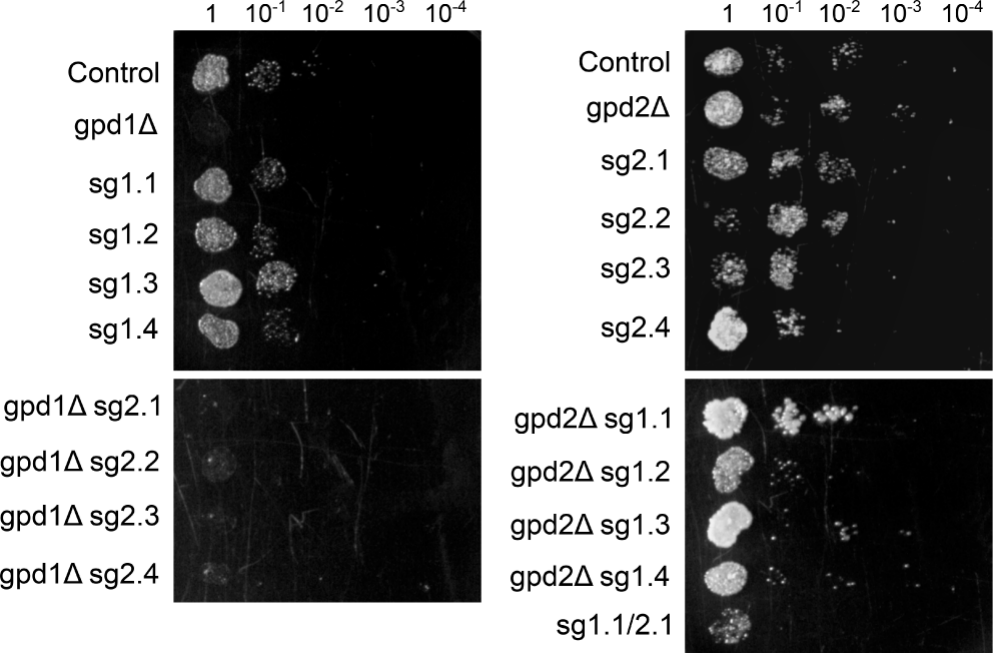
Growth of *S. cerevisiae* serial diluted cultures of the strains carrying sole CRISPRi modulation and CRISPRi modulation combined with single deletion compared to the control, gpd1Δ, and gpd2Δ strains under osmotic stress (solid YPD medium with 25% glucose) after 72 h of incubation at 30 °C.

As already reported in other studies, the control and gpd2Δ strain grew well under osmotic stress in solid medium.^26^ Also, the gpd1Δ strain could not grow in the tested condition, since its expression is crucial for cell viability in such conditions. Interestingly, among the strains solely modulated by CRISPRi, only the sg1.1/2.1 strain presented hindered growth compared to the control, even though minor impacts can be noted in the sg2.3 and sg2.4 strains’ growth (Figure 6). The strains carrying both single gene deletion and dCas9-Mxi1 expression vectors showed distinct growth phenotypes according to the deleted *GPD* paralog. The gpd1Δ-based strains did not grow, without any distinction between the different sites targeted for *GPD2* repression. The opposite was observed for the gpd2Δ-based strains, which showcase growth in high osmolarity regardless of the targeted site in the *GPD1* promoter (Figure 6).

Although promising, the results obtained from growth in solid medium are not completely representative of the strains’ fermentative performance, since it does not take into account important variables such as aeration and stirring. Thus, to deepen our understanding of the sole CRISPRi-mediated modulation potential of generating osmosensitive cells, we selected the *GPD1*-modulated strains to perform a very high-gravity (VHG) fermentation in 25% glucose YPD medium. The *GPD1*-modulated strains were chosen because of the *GPD1*-specific role in the yeast cell response to high osmolarity. The time course graphs of cell growth, glucose consumption, glycerol, and ethanol production throughout the 72 h of VHG fermentation, together with the glycerol and ethanol yield values, are available in Figure S4 and Table S7. Biomass values, glycerol final concentrations, ethanol final concentrations, and SEP are also listed in Table S8.

The glycerol production by the *GPD1*-modulated strains followed a distinct pattern in VHG fermentation compared with standard fermentation. The sg1.1 strain showed a 3-fold glycerol production reduction compared to the control. This reduction is almost the same observed for gpd1Δ strain and sg1.2 strain. In turn, the sg1.3 strain produced only 33% less glycerol than the control, while the sg1.4 strain produced almost as much glycerol as the control strain (Figure 7A). However, regarding biomass formation, the sg1.3 strain grew almost as much as the control and the sg1.4 strain grew 40.9% more, while gpd1Δ, sg1.1, and sg1.2 showed a 2–4-fold growth decrease (Figure 7B).

**Figure 7:**
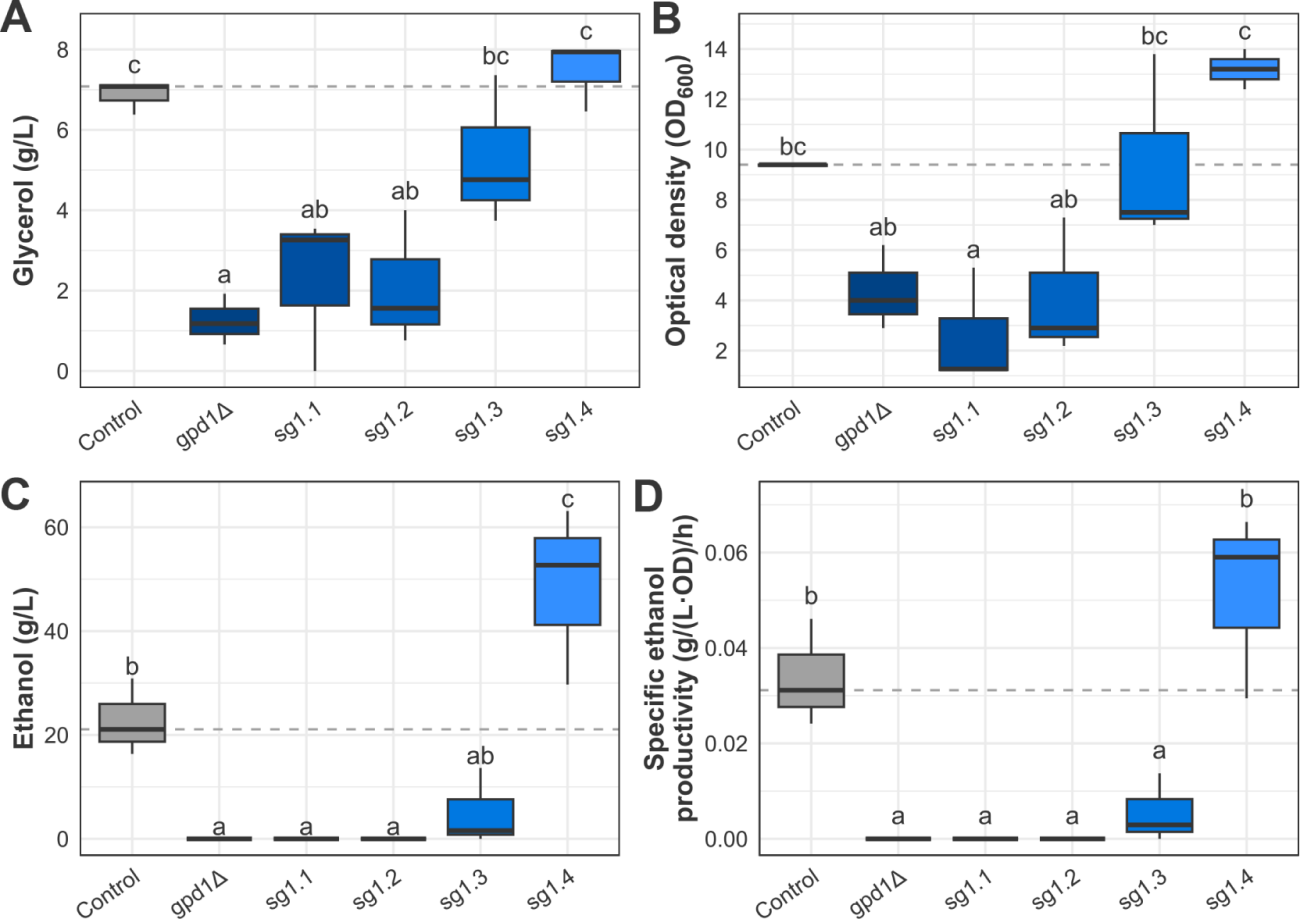
Fermentative profile of the *GPD1-*modulated strains after 72 h of very high-gravity (VHG) fermentation in YPD 25% glucose deepens the understanding of the strains’ phenotypes in a high-osmolarity environment. A. Glycerol final concentration for each of the tested strains. B. Biomass formation of the tested strains according to the optical density at 600 nm of the culture. C. Ethanol final concentration of the tested strains. D. Specific ethanol productivity of each strain. Boxplots represent minimum, maximum, quartile, and median values, and the letters indicate statistically significant differences between strains.

Interestingly, the sg1.4 strain was the only *GPD1*-modulated strain that produced more ethanol than the control, reaching 48.51 g/L and a 52.9% higher SEP value (Figure 7C and D). Although unexpected, the sg1.4 strain fermentative profile in VHG fermentation is somehow equivalent to the sg1.1 profile in standard fermentation. These results shed light on a carbon drift dynamic that is more complex than postulated in glycerol production reduction studies. Moreover, the hampered growth of the sg1.1 and sg1.2 strains in contrast to their apparent normal growth in solid medium (Figures 6 and 7B) highlights the sharp difference between liquid and solid cultures’ phenotypes because of the specific conditions observed in each experiment.

In light of our results, the application of dCas9-mediated gene regulation is taking its place as a promising alternative for metabolic engineering. Applying CRISPRi to the glycerol synthetic pathway, target-site-dependent glycerol production reduction was obtained without genome sequence alterations. We could also engineer a promising osmoresistant strain with increased ethanol productivity in semianaerobic conditions. Moreover, our results shed light on unexpected dynamics between biomass formation, ethanol, and glycerol production in relation to each *GPD* paralog modulation. Therefore, the application of fine-tuning techniques in metabolic engineering is useful not only to alter metabolite production levels but also to explore the dynamics between metabolism, gene expression, and cell physiology.

Finally, we highlight the need for further investigation into the expression patterns of *GPD* genes and the impact of their modulation in cell transcriptomics to better understand the distinct consequences of CRISPRi in each paralog. In our study, we focused on studying the application of CRISPRi in the glycerol synthesis pathway and characterizing the fermentative profiles of CRISPRi-modulated strains. With this goal, we chose a laboratory strain as a genetic background and a plasmid-based strategy. However, further steps include *GPD* expression fine-tuning applied to industrial *S. cerevisiae* strains and the development of more stable genetic modifications to improve the feasibility of the approach such as integration of the genes needed for CRISPRi proper function.

## Supplementary Material



## Notes

Additional experimental material, methods, and results of fermentation and growth assays (PDF)
