# Supplementary material for: Impact of CRISPRi-Mediated Titration of GPD Genes on the Fermentative Performance of S. cerevisiae
Source: ACS Synth Biol. 2025 Oct 16;14(11):4412–23. doi: 10.1021/acssynbio.5c00316 (PMC12645569; doi:10.1021/acssynbio.5c00316)
Supplement: Supplementary file 1 [file sb5c00316_si_001.pdf]

SUPPLEMENTARY INFORMATION

CRISPRi-Mediated Titration of *GPD* Genes in *S. cerevisiae* Enhances Fermentative Performance

João Miguel Spavieri[a], Thiago Gaspar Inacio[a], Gustavo Seguchi[a], Brenda Cristina de Souza[a], Gonçalo A. G. Pereira[a,*], Fellipe de Mello[a]

[a]Departamento de Genética, Evolução e Bioagentes, UNICAMP, Campinas, SP, Brazil

[*] Corresponding author: goncalo@unicamp.br

Supplementary Tables

Table S1: Selected target sites sequences, localization, corresponding sgRNA, nucleosome occupancy index and chromatin accessibility index according to Yeast CRISPRi web tool[1].

Target gene	sgRNA	Localization	Sequence (5' → 3')	Nucleosome occupancy	Chromatin accessibility
GPD1	sg1.1	-140	TAACGTAACAGTTTCACTCA	0,18	0,14
	sg1.2	-266	AGGGACAAAATAGGGAAAAC	0,08	0,23
	sg1.3	-351	GCGCCCCGTTTACGTATTGG	0,07	0,98
	sg1.4	-492	AGAAAACAGAAGGCCAAGAC	0,06	0,36
GPD2	sg2.1	-130	AAGAACCCAAGGATAAATCA	0,08	0,12
	sg2.2	-274	CTGCTGAACAATAGATTAAT	0	0,28
	sg2.3	-340	GAGGTGCCGATGGGTTGCTG	0,18	0,3
	sg2.4	-480	GAAGATACACGCTTGCATGA	0,32	0,38

Table S2: Oligonucleotides and primers used in the study.

Name		Sequence (5' → 3')	Function
JMO007	F	ggcgccggctgggcaacaccttcgggtggcgaatgggact TAACGTAACAGTTTCACTCAgttttagagctaga aatagcaagttaaaataaggctagtc	ssOligos for hybridization of sg1.1 dsOligo used for cloning sg1.1 sequence in dCas9-Mxi1 vector
JMO008	R	gactagccttattttaacttgctatttctagctctaaaacTGAG TGAAACTGTTACGTTAagtcccattcgccacccga aggtgttgcccagccggcgcc	
JMO009	F	ggcgccggctgggcaacaccttcgggtggcgaatgggact AGGGACAAAATAGGGAAAACgttttagagctag aaatagcaagttaaaataaggctagtc	ssOligos for hybridization of sg1.2 dsOligo used for cloning sg1.2 sequence in dCas9-Mxi1 vector
JMO010	R	gactagccttattttaacttgctatttctagctctaaaacGTTT TCCCTATTTTGTCCCTagtcccattcgccacccga aggtgttgcccagccggcgcc	
JMO011	F	ggcgccggctgggcaacaccttcgggtggcgaatgggact GCGCCCCGTTTACGTATTGGgttttagagctag aaatagcaagttaaaataaggctagtc	ssOligos for hybridization of sg1.3 dsOligo used for cloning sg1.3 sequence in dCas9-Mxi1 vector
JMO012	R	gactagccttattttaacttgctatttctagctctaaaacCCAA TACGTAAACGGGGCGCagtcccattcgccacccg aaggtgttgcccagccggcgcc	
JMO013	F	ggcgccggctgggcaacaccttcgggtggcgaatgggact AGAAAACAGAAGGCCAAGACgttttagagctag aaatagcaagttaaaataaggctagtc	ssOligos for hybridization of sg1.4 dsOligo used for cloning sg1.4 sequence in dCas9-Mxi1 vector
JMO014	R	gactagccttattttaacttgctatttctagctctaaaacGTCT TGGCCTTCTGTTTTCTagtcccattcgccacccga aggtgttgcccagccggcgcc	
JMO015	F	ggcgccggctgggcaacaccttcgggtggcgaatgggact AAGAACCCAAGGATAAATCAgttttagagctaga aatagcaagttaaaataaggctagtc	ssOligos for hybridization of sg2.1 dsOligo used for cloning sg2.1 sequence in dCas9-Mxi1 vector
JMO016	R	gactagccttattttaacttgctatttctagctctaaaacTGAT TTATCCTTGGGTTCTTagtcccattcgccacccga aggtgttgcccagccggcgcc	
JMO017	F	ggcgccggctgggcaacaccttcgggtggcgaatgggact CTGCTGAACAATAGATTAATgttttagagctaga aatagcaagttaaaataaggctagtc	ssOligos for hybridization of sg2.2 dsOligo used for cloning sg2.2 sequence in dCas9-Mxi1 vector
JMO018	R	gactagccttattttaacttgctatttctagctctaaaacATTA ATCTATTGTTCAGCAGagtcccattcgccacccga aggtgttgcccagccggcgcc	
JMO019	F	ggcgccggctgggcaacaccttcgggtggcgaatgggact GAGGTGCCGATGGGTTGCTGgttttagagctag aaatagcaagttaaaataaggctagtc	ssOligos for hybridization of sg2.3 dsOligo used for cloning sg2.3 sequence in dCas9-Mxi1 vector

JMO020	R	gactagccttattttaacttgctatttctagctctaaaacCAGCAACCCATCGGCACCTCagtcccattcgccacccgaaggtgttgcccagccggcgcc	
JMO021	F	ggcgccggctgggcaacaccttcgggtggcgaatgggactGAAGATACACGCTTGCATGAgttttagagctagaaatagcaagttaaaataaggctagtc	ssOligos for hybridization of sg2.4 dsOligo used for cloning sg2.4 sequence in dCas9-Mxi1 vector
JMO022	R	gactagccttattttaacttgctatttctagctctaaaacTCATGCAAGCGTGTATCTTCagtcccattcgccacccgaaggtgttgcccagccggcgcc	
JMO023	F	ACCCCCCCCCTCCACAAACACAAATATTGATAATATAAAGgacatggaggcccagaatac	Primers for amplification of hphMX6 from pAG32[2] with homologous ends for *GPD1* knock-in
JMO024	R	AGTGGGGGAAAGTATGATATGTTATCTTTCTCCAATAAATcagtatagcgaccagcattc	
JMO025	F	CTCTTTCCCTTTCCTTTTCCTTCGCTCCCCTTCCTTATCAgacatggaggcccagaatac	Primers for amplification of hphMX6 from pAG32[2] with homologous ends for *GPD2* knock-in
JMO026	R	GCAACAGGAAAGATCAGAGGGGGAGGGGGGGGAGAGTGTcagtatagcgaccagcattc	
GUO037	F	ACTGTGACTAGTatgtgcttcagtattacatttttg	Amplification of the sg1.1 expression cassette from the dCas9-Mxi1 (sg1.1) vector with ends homologous to *KanMX* and dCas9-Mxi1 vector backbone for multiplex plasmid recombination
GUO038	R	GACTCTCTGCAGtatccactagacagaagtttgc	
GUO039	F	gctgagcagttacagagatgttacgaac	Amplification of the *KanMX* cassette from dCas9-Mxi1 backbone vector with ends homologous to sg2.1 expression cassette and sg1.1 expression cassette for multiplex plasmid recombination
GUO040	R	agcacatACTAGTCACAGTtccagtatagcgaccagcattcac	
GUO041	F	tggataCTGCAGAGAGTCgtatcacgtgctataaaaataatt	Amplification of the dCas9-Mxi1 vector backbone with ends homologous to sg1.1 expression cassette and dCas9-Mxi1 expression cassette for multiplex plasmid recombination
GUO042	R	cgttcgtctgcaattatc	
BC013	F	ATGCAAACCGCTGCTCAA	Amplification of *ACT1* from cDNA as reference gene for qPCR assays (designed by Yang et al.[3])
BC014	R	AGTTTGGTCAATACCGGCAGA	
JMO040	F	GCGAGGGCAAGGACGTCGAC	Amplification of *GPD1* from cDNA for qPCR assays (designed by Noti et al.[4])
JMO041	R	TGGATGGCAGCAGAAGCGTTGT	

JMO042	F	TTTCCCAGAATCCAAAGTCG	Amplification of *GPD2* from cDNA for qPCR assays (designed by Noti et al.[4])
JMO043	R	CTGAGCAGGTGGTGATCAGA	

Table S2: List of plasmids.

Name	Description	Origin
EC2_3_dCas9_Mxi1_sgRNA	dCas9-Mxi1 low copy plasmid without sgRNA sequence.	Cámara et al.[5]
dCas9_Mxi1_sg1.1	dCas9-Mxi1 low copy plasmid with sg1.1 sequence.	This study
dCas9_Mxi1_sg1.2	dCas9-Mxi1 low copy plasmid with sg1.2 sequence.	This study
dCas9_Mxi1_sg1.3	dCas9-Mxi1 low copy plasmid with sg1.3 sequence.	This study
dCas9_Mxi1_sg1.4	dCas9-Mxi1 low copy plasmid with sg1.4 sequence.	This study
dCas9_Mxi1_sg2.1	dCas9-Mxi1 low copy plasmid with sg2.1 sequence.	This study
dCas9_Mxi1_sg2.2	dCas9-Mxi1 low copy plasmid with sg2.2 sequence.	This study
dCas9_Mxi1_sg2.3	dCas9-Mxi1 low copy plasmid with sg2.3 sequence.	This study
dCas9_Mxi1_sg2.4	dCas9-Mxi1 low copy plasmid with sg2.4 sequence.	This study
dCas9_Mxi1_sg1.1_2.1	dCas9-Mxi1 low copy plasmid with sg1.1 and sg2.1 sequence.	This study

Table S4: List of yeast strains.

Name	Relevant genotype	Origin
BY4742	*MATα his3Δ1 leu2Δ0 lys2Δ0 ura3Δ0*	Goldstein and McCusker[2]
Control	BY4742 containing EC2_3_dCas9_Mxi1_sgRNA	This study
gpd1Δ	BY4742 gpd1::hphMX6 containing EC2_3_dCas9_Mxi1_sgRNA	This study
gpd2Δ	BY4742 gpd2::hphMX6 containing EC2_3_dCas9_Mxi1_sgRNA	This study
sg1.1	BY4742 containing dCas9_Mxi1_sg1.1	This study

sg1.2	BY4742 containing dCas9_Mxi1_sg1.2	This study
sg1.3	BY4742 containing dCas9_Mxi1_sg1.3	This study
sg1.4	BY4742 containing dCas9_Mxi1_sg1.4	This study
sg2.1	BY4742 containing dCas9_Mxi1_sg2.1	This study
sg2.2	BY4742 containing dCas9_Mxi1_sg2.2	This study
sg2.3	BY4742 containing dCas9_Mxi1_sg2.3	This study
sg2.4	BY4742 containing dCas9_Mxi1_sg2.4	This study
sg1.1/2.1	BY4742 containing dCas9_Mxi1_sg1.1_2.1	This study
gpd2Δ sg1.1	BY4742 gpd2::hphMX6 containing dCas9_Mxi1_sg1.1	This study
gpd2Δ sg1.2	BY4742 gpd2::hphMX6 containing dCas9_Mxi1_sg1.2	This study
gpd2Δ sg1.3	BY4742 gpd2::hphMX6 containing dCas9_Mxi1_sg1.3	This study
gpd2Δ sg1.4	BY4742 gpd2::hphMX6 containing dCas9_Mxi1_sg1.4	This study
gpd1Δ sg2.1	BY4742 gpd1::hphMX6 containing dCas9_Mxi1_sg2.1	This study
gpd1Δ sg2.2	BY4742 gpd1::hphMX6 containing dCas9_Mxi1_sg2.2	This study
gpd1Δ sg2.3	BY4742 gpd1::hphMX6 containing dCas9_Mxi1_sg2.3	This study
gpd1Δ sg2.4	BY4742 gpd1::hphMX6 containing dCas9_Mxi1_sg2.4	This study

Table S5: Glycerol yield ($Y_{glycerol}$) and ethanol yield ($Y_{ethanol}$) after 24 hours of fermentation.

Strain	$Y_{Glycerol}$ ($g_{Glycerol}/g_{Glucose}$)	$Y_{Ethanol}$ ($g_{Ethanol}/g_{Glucose}$)
**Control**	0,018 ± 0,001	0,477 ± 0,010
**gpd1Δ**	0,011 ± 0,006	0,483 ± 0,010
**gpd2Δ**	0,009 ± 0,002	0,480 ± 0,007
**sg1.1**	0,021 ± 0,002	0,492 ± 0,010
**sg1.2**	0,014 ± 0,001	0,459 ± 0,013
**sg1.3**	0,016 ± 0,003	0,459 ± 0,002
**sg1.4**	0,018 ± 0,002	0,473 ± 0,006
**sg2.1**	0,015 ± 0,001	0,473 ± 0,002
**sg2.2**	0,017 ± 0,002	0,469 ± 0,009
**sg2.3**	0,019 ± 0,002	0,468 ± 0,009
**sg2.4**	0,021 ± 0,002	0,451 ± 0,012

**sg1.1/2.1**	0,017 ± 0,002	0,463 ± 0,027
**gpd2Δ sg1.1**	0,008 ± 0,002	0,466 ± 0,014
**gpd2Δ sg1.2**	0,009 ± 0,002	0,461 ± 0,011
**gpd2Δ sg1.3**	0,006 ± 0,001	0,458 ± 0,006
**gpd2Δ sg1.4**	0,007 ± 0,000	0,457 ± 0,010
**gpd1Δ sg2.1**	0,005 ± 0,002	0,461 ± 0,004
**gpd1Δ sg2.2**	0,008 ± 0,001	0,450 ± 0,018
**gpd1Δ sg2.3**	0,006 ± 0,002	0,463 ± 0,018
**gpd1Δ sg2.4**	0,007 ± 0,001	0,461 ± 0,020

Table S6: Biomass, glycerol concentration, ethanol concentration, and specific ethanol productivity (SEP) after 24 hours of fermentation.

**Strain**	**Biomass** (OD_{600})	**Glycerol** (g/L)	**Ethanol** (g/L)	**SEP** ($g_{Ethanol}$/(L·OD)/h)
**Control**	10,45 ± 0,343	0,854 ± 0,036	22,4 ± 0,457	0,089 ± 0,002
**gpd1Δ**	10,60 ± 0,340	0,503 ± 0,263	22,6 ± 0,470	0,089 ± 0,005
**gpd2Δ**	11,23 ± 0,083	0,413 ± 0,061	22,5 ± 0,312	0,084 ± 0,001
**sg1.1**	10,53 ± 0,273	0,980 ± 0,076	23,1 ± 0,479	0,091 ± 0,001
**sg1.2**	9,49 ± 0,369	0,657 ± 0,054	21,5 ± 0,621	0,095 ± 0,001
**sg1.3**	10,01 ± 0,367	0,736 ± 0,124	21,5 ± 0,084	0,090 ± 0,004
**sg1.4**	10,67 ± 0,264	0,836 ± 0,076	22,2 ± 0,266	0,087 ± 0,002
**sg2.1**	10,65 ± 0,170	0,716 ± 0,046	22,2 ± 0,120	0,087 ± 0,001
**sg2.2**	11,08 ± 0,519	0,782 ± 0,081	22,0 ± 0,392	0,083 ± 0,003
**sg2.3**	10,53 ± 0,583	0,861 ± 0,105	22,0 ± 0,442	0,087 ± 0,005
**sg2.4**	10,41 ± 0,110	0,966 ± 0,092	21,2 ± 0,583	0,085 ± 0,003
**sg1.1/2.1**	10,04 ± 0,111	0,784 ± 0,084	21,7 ± 1,278	0,090 ± 0,006
**gpd2Δ sg1.1**	8,87 ± 0,323	0,354 ± 0,065	21,9 ± 0,657	0,103 ± 0,002
**gpd2Δ sg1.2**	9,77 ± 0,153	0,429 ± 0,092	21,6 ± 0,493	0,092 ± 0,003
**gpd2Δ sg1.3**	10,22 ± 0,312	0,280 ± 0,054	21,5 ± 0,273	0,088 ± 0,003
**gpd2Δ sg1.4**	9,95 ± 0,117	0,337 ± 0,015	21,4 ± 0,473	0,090 ± 0,003
**gpd1Δ sg2.1**	10,44 ± 0,334	0,228 ± 0,090	21,6 ± 0,194	0,086 ± 0,004
**gpd1Δ sg2.2**	11,24 ± 0,884	0,360 ± 0,015	21,1 ± 0,870	0,078 ± 0,005
**gpd1Δ sg2.3**	10,60 ± 0,178	0,279 ± 0,082	21,7 ± 0,838	0,085 ± 0,004
**gpd1Δ sg2.4**	10,69 ± 0,598	0,305 ± 0,034	21,6 ± 0,910	0,084 ± 0,005

Table S6: Glycerol yield ($Y_{glycerol}$) and ethanol yield ($Y_{ethanol}$) after 72 hours of Very High-Gravity (VHG) fermentation.

Strain	$Y_{Glycerol}$ ($g_{Glycerol}/g_{Glucose}$)	$Y_{Ethanol}$ ($g_{Ethanol}/g_{Glucose}$)
**Control**	0,065 ± 0,007	0,219 ± 0,086
**gpd1Δ**	0,033 ± 0,015	0,000
**sg1.1**	0,043 ± 0,039	0,000
**sg1.2**	0,039 ± 0,025	0,000
**sg1.3**	0,066 ± 0,018	0,059 ± 0,087
**sg1.4**	0,047 ± 0,002	0,298 ± 0,071

Table S7: Biomass, glycerol concentration, ethanol concentration, and specific ethanol productivity (SEP) after 72 hours of VHG fermentation.

Strain	**Biomass** (OD_{600})	**Glycerol** (g/L)	**Ethanol** (g/L)	**SEP** ($g_{Ethanol}$/(L·OD)/h)
**Control**	9,37 ± 0,058	6,87 ± 0,423	22,76 ± 7,40	0,034 ± 0,011
**gpd1Δ**	4,37 ± 1,680	1,25 ± 0,633	0,00	0,000
**sg1.1**	2,59 ± 2,350	2,27 ± 1,968	0,00	0,000
**sg1.2**	4,13 ± 2,768	2,11 ± 1,688	0,00	0,000
**sg1.3**	9,43 ± 3,790	5,29 ± 1,867	5,07 ± 7,46	0,006 ± 0,007
**sg1.4**	13,20 ± 0,800	7,46 ± 0,866	48,51 ± 17,10	0,052 ± 0,020

Supplementary Figures

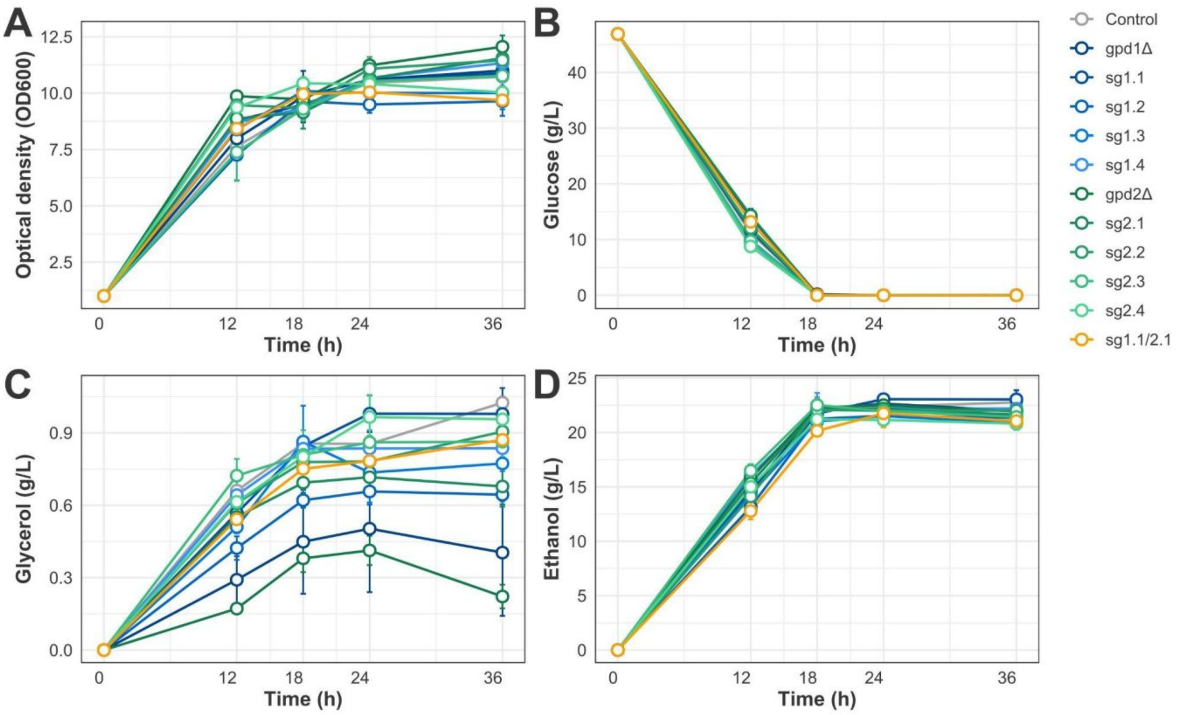

Figure S1. Cell density, glucose concentration, glycerol concentration, and ethanol concentration by the CRISPRi-modulated, single-knockout, and control strains during semi-anaerobic batch-culture fermentation in YPD medium supplemented with geneticin at 30 ºC and 200 rpm throughout 36 hours. Samples were analyzed at the beginning of the process (time 0), and after 12, 18, 24, and 36 hours of fermentation. Mean and standard deviation values calculated from triplicates are shown. **A.** Culture cell density (OD_{600}) time course over the 36-hour fermentation for each CRISPRi-modulated, single-knockout, and control strain. **B.** Glucose concentration (g/L) time course over the 36-hour fermentation for each CRISPRi-modulated, single-knockout, and control strain. **C.** Glycerol concentration (g/L) time course over the 36-hour fermentation for each CRISPRi-modulated, single-knockout, and control strain. **D.** Ethanol concentration (g/L) time course over the 36-hour fermentation for each CRISPRi-modulated, single-knockout, and control strain.

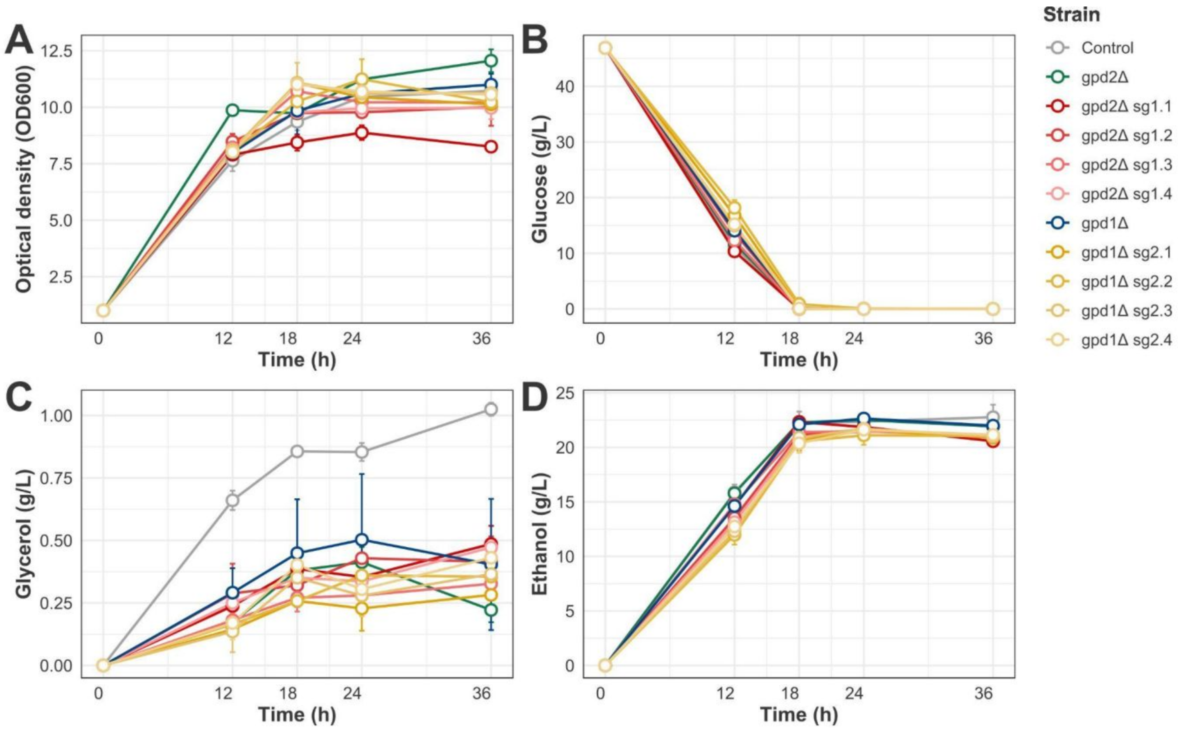

Figure S2. Cell density, glucose concentration, glycerol concentration, and ethanol concentration by the *GPD* concurrent knockout and CRISPRi modulation, single-knockout, and control strains during semi-anaerobic batch-culture fermentation in YPD medium supplemented with geneticin at 30 ºC and 200 rpm throughout 36 hours. Samples were analyzed at the beginning of the process (time 0), and after 12, 18, 24, and 36 hours of fermentation. Mean and standard deviation values calculated from triplicates are shown. **A.** Culture cell density (OD_{600}) time course over the 36-hour fermentation for each *GPD* concurrent knockout and CRISPRi modulation, single-knockout, and control strain. **B.** Glucose concentration (g/L) time course over the 36-hour fermentation for each *GPD* concurrent knockout and CRISPRi modulation, single-knockout, and control strain. **C.** Glycerol concentration (g/L) time course over the 36-hour fermentation for each *GPD* concurrent knockout and CRISPRi modulation, single-knockout, and control strain. **C.** Ethanol concentration (g/L) time course over the 36-hour fermentation for each *GPD* concurrent knockout and CRISPRi modulation, single-knockout, and control strain.

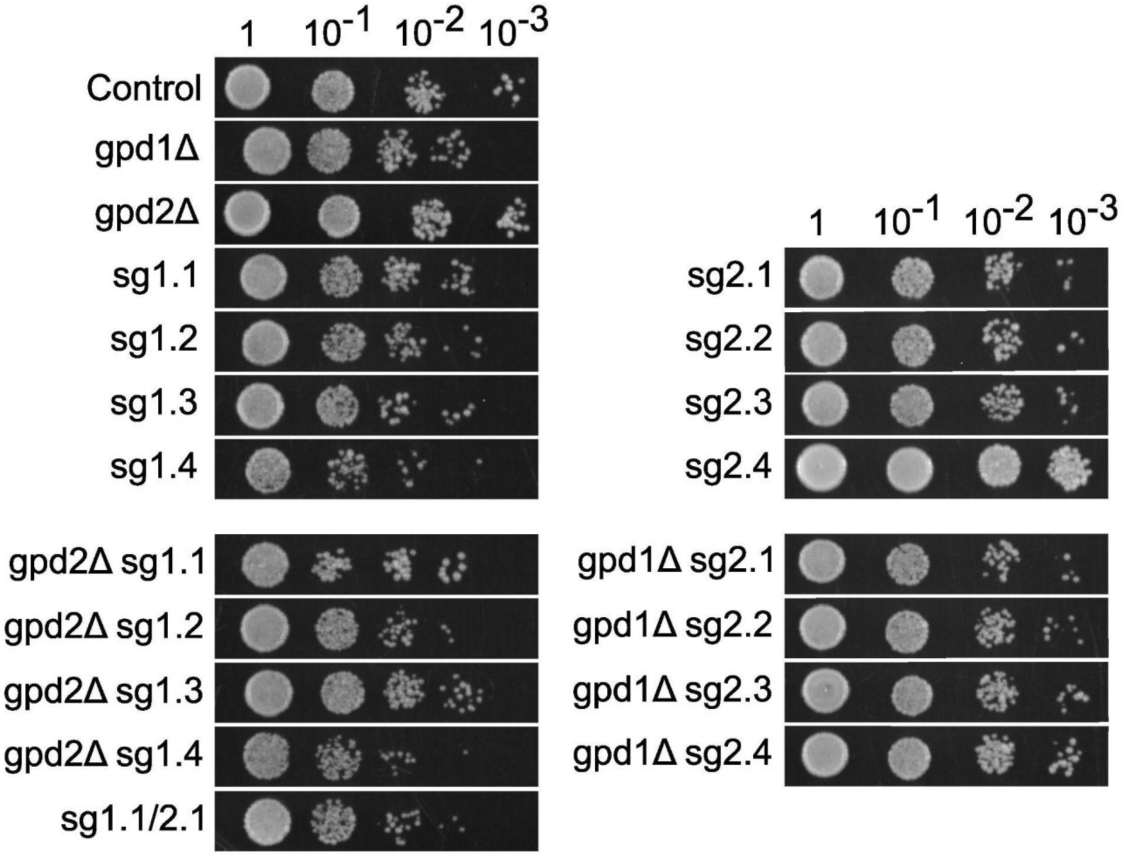

Figure S3. Growth of serial diluted cultures of the strains in solid YPD (2% glucose) medium after 48h hours of incubation at 30 ºC.

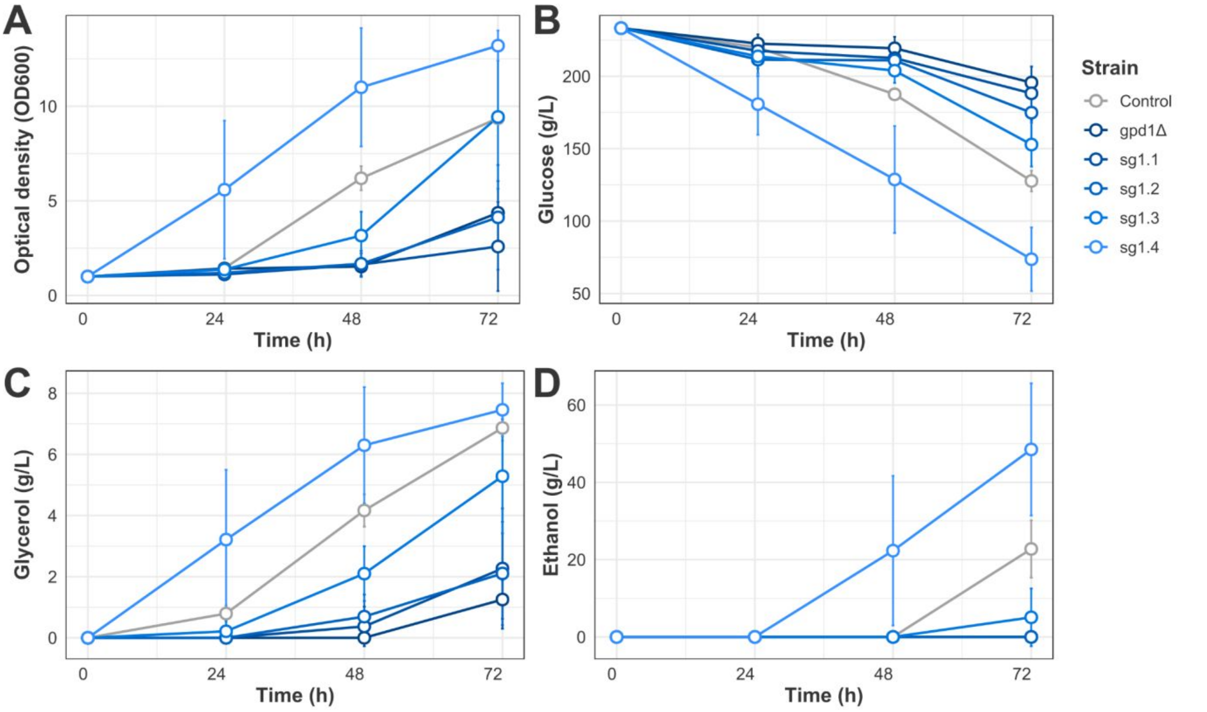

Figure S4. Cell density, glucose concentration, glycerol concentration, and ethanol concentration by the *GPD1*-modulated, gpd1Δ and control strains during Very High-Gravity

(VHG) batch-culture fermentation in 25% glucose YPD medium supplemented with geneticin at 30 ºC and 200 rpm throughout 72 hours. Samples were analyzed at the beginning of the process (time 0), and after 24, 48, and 72 hours of fermentation. Mean and standard deviation values calculated from triplicates are shown. **A.** Culture cell density (OD_{600}) time course over the 72-hour fermentation for each strain. **B.** Glucose concentration (g/L) time course over the 72-hour fermentation for each strain. **C.** Glycerol concentration (g/L) time course over the 72-hour fermentation for each strain. **C.** Ethanol concentration (g/L) time course over the 72-hour fermentation for each strain.

REFERENCES

(1) Smith, J. D.; Suresh, S.; Schlecht, U.; Wu, M.; Wagih, O.; Peltz, G.; Davis, R. W.; Steinmetz, L. M.; Parts, L.; St.Onge, R. P. Quantitative CRISPR Interference Screens in Yeast Identify Chemical-Genetic Interactions and New Rules for Guide RNA Design. *Genome Biol.* **2016**, *17* (1), 45. https://doi.org/10.1186/s13059-016-0900-9.

(2) Goldstein, A. L.; McCusker, J. H. Three new dominant drug resistance cassettes for gene disruption in Saccharomyces cerevisiae. *Yeast* **1999**, *15* (14), 1541–1553. https://doi.org/10.1002/(SICI)1097-0061(199910)15:14<1541::AID-YEA476>3.0.CO;2-K.

(3) Yang, B.-X.; Xie, C.-Y.; Xia, Z.-Y.; Wu, Y.-J.; Gou, M.; Tang, Y.-Q. Improving Xylitol Yield by Deletion of Endogenous Xylitol-Assimilating Genes: A Study of Industrial Saccharomyces Cerevisiae in Fermentation of Glucose and Xylose. *FEMS Yeast Res.* **2021**, *20* (8), foaa061. https://doi.org/10.1093/femsyr/foaa061.

(4) Noti, O.; Vaudano, E.; Pessione, E.; Garcia-Moruno, E. Short-Term Response of Different *Saccharomyces Cerevisiae* Strains to Hyperosmotic Stress Caused by Inoculation in Grape Must: RT-qPCR Study and Metabolite Analysis. *Food Microbiol.* **2015**, *52*, 49–58. https://doi.org/10.1016/j.fm.2015.06.011.

(5) Cámara, E.; Lenitz, I.; Nygård, Y. A CRISPR Activation and Interference Toolkit for Industrial Saccharomyces Cerevisiae Strain KE6-12. *Sci. Rep.* **2020**, *10* (1), 14605. https://doi.org/10.1038/s41598-020-71648-w.
